# Optical Extraction of Single Microplastics Followed by Online Molecular and Elemental Characterization

**DOI:** 10.1021/acs.analchem.5c05030

**Published:** 2026-01-26

**Authors:** Matthias Elinkmann, Christian Neuper, Manuel Candussi, Raquel Gonzalez de Vega, Svenja B. Seiffert, Patrizia M. Schmidt, Harald Fitzek, Christian Hill, David Clases

**Affiliations:** ◪ NanoMicroLab, Institute of Chemistry, 27267 University of Graz, 8010 Graz, Austria; ‡ Brave Analytics GmbH, 8010 Graz, Austria; § TESLA, Institute of Chemistry, University of Graz, 8010 Graz, Austria; ∥ BASF SE, Department of Chemical, Material & Regulatory Science, 67056 Ludwigshafen, Germany; ⊥ Graz Centre for Electron Microscopy, Steyrergasse 17, 8010 Graz, Austria; # Gottfried Schatz Research Center, Medical Physics and Biophysics, Medical University of Graz, 8010 Graz, Austria

## Abstract

The accurate characterization of microplastics (MPs) in complex matrices remains a major analytical challenge and requires advanced methods, which decipher information on size and polymer identity at single particle resolution. Single particle (SP) inductively coupled plasma–mass spectrometry (ICP-MS) has emerged as an element-selective method to detect individual particles in a one-by-one fashion and can be used to detect and characterize MPs regarding carbon mass and particle sizes. However, this technique has two relevant shortcomings. First, its ability to pinpoint small MPs requires a low dissolved C-background. Second, SP ICP-MS neither distinguishes different MP polymer species from each other nor from other C-particulates (e.g., cells, black carbon). As such, the application of SP ICP-MS is significantly limited when targeting MPs in complex matrices without *a priori* knowledge. Here, we present a novel trimodal analytical platform that integrates optofluidic force induction (OF2i), single particle Raman spectroscopy (SP Raman), and SP ICP-MS for multimodal online MP characterization. The main objective was the development and demonstration of an optical extraction mechanism, in which an optical trap employing a weakly focused laser vortex beam was used to immobilize MPs from a sample suspension. This provided two analytical opportunities, which were demonstrated in conjunction with a SP Raman module and SP ICP-MS. First, the optical trapping enabled the investigation of inelastically scattered light of individual MPs via Raman spectroscopy and consequently, the identification of polymer type. Second, the trapping enabled a matrix exchange, reducing the background signal in SP ICP-MS. Both the polymer identification and the reduction of background signal resulted in improved detection and calibration capabilities in SP ICP-MS. Polymer type identification via SP Raman provided the C-mass fraction and polymer density in MPs, which are critical factors for size estimations in SP ICP-MS. However, the reduced background and improved size detection limit enabled the analysis of smaller MPs. In a proof-of-concept, 5 μm polystyrene particles were dispersed in a high carbon content matrix (1 g C/L) and analyzed via OF2i-SP Raman-SP ICP-MS using the optical extraction mechanism. The mass and size detection limits were improved by factors of 28 and 3.1, respectively, and were determined to be 1.0 μm and 0.6 pg of carbon per particle. In a second proof-of-concept, polyamide-6 (PA-6) MPs were spiked into soil to simulate a complex terrestrial environment at the lab-scale. Following the resuspension, PA-6 MPs were optically extracted and analyzed using SP Raman and SP ICP-MS. The optical extraction of MPs is a new concept, which enables isolation of specific polymer particles for molecular and elemental single particle characterization. However, the developed methods can also be expanded to characterize inorganic (non-polymeric) nano- and microstructures.

## Graphical abstract

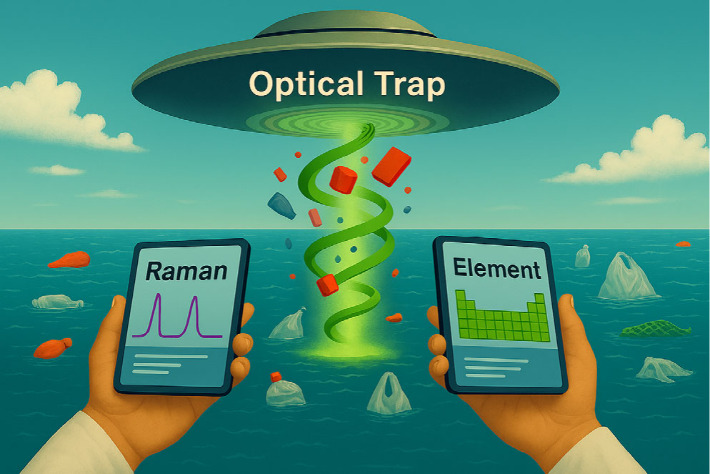

## Introduction

Microplastics (MPs) have emerged as pervasive environmental pollutants and can be found ubiquitously in both terrestrial and aquatic systems.^1,2^ They are typically defined as solid polymer particles smaller than 5 mm in size and are either manufactured at these sizes for specific applications (primary MPs) or originate from the breakdown of macroplastic (secondary MPs).^3–5^ Their impact on both ecosystems and health has raised major concerns, which sparked analytical endeavors to develop dedicated methods for tracing and thoroughly characterizing these particles.^6,7^ However, MPs consist of varying polymer types, have broad size distributions, and are contained in complex environmental or biological matrices alongside a plethora of natural colloidal structures. Currently, there is a significant analytical gap, and adequate technology and methodology providing high selectivity and sensitivity are required.^8–11^

Many established methods offer ensemble analysis without granting access to single particles and/or are not capable of counting small MPs at environmental/biological concentrations sufficiently fast to gain a statistically meaningful perspective on different polymer species. For example, methods like dynamic light scattering do not offer sufficient selectivity to distinguish MPs from natural colloids and cannot identify different polymer types.^6^ Visual analysis with techniques such as electron microscopy enable the analysis of individual particles but are not applicable to pinpoint MPs at natural levels, to identify their polymer type, and to detect them in natural matrices. Consequently, they are often unable to collect meaningful data on the mixing states of MPs in the environment.^13,14^ However, FTIR and Raman microscopy^12^ may be applicable to identify polymer species in isolated MPs but can be time-consuming and limited in their ability to count sufficient particles in complex matrices -especially at low PNCs and with low diameters.^6^

A relatively new method for the analysis of MPs is inductively coupled plasma–mass spectrometry (ICP-MS) which can be operated in “single particle” (SP) mode to detect particles individually. ICP-MS instrumentation can be equipped with different mass analyzers: While quadrupoles offer high sensitivity for only one selected *m*/*z* in single particle mode, TOF analyzers can analyze virtually all elements in a particle and further enable non-target particle screenings.^15,16^ When targeting C, both ICP-TOFMS as well as ICP-QMS are applicable to the analysis of single MPs even in complex matrices.^17,18^ However, it is worth noting that all species/molecular information contained in MPs (e.g., polymer type) is lost during the hard atomization and ionization process in the ICP. As such, it is not possible to discriminate specific particulate C species and the analysis of samples containing for example different MPs, particulate organic matter, cells, bacteria and/or black carbon is very limited.^19^ Furthermore, the detection limit for SP analysis of MPs is capped by the C background from dissolved species. The mean background intensity determines a critical limit-based threshold over which a signal is identified as a SP. At high background, detection limits therefore increase. Finally, characterizing important SP parameters such as particle transport efficiency becomes increasingly difficult when analyzing particles at the microscale, and it appears that large particles are underestimated when assessing both number concentrations and size distributions.^20^

Advancing MP analysis is possible when introducing and hyphenating complementary techniques, which can decipher polymer identity while providing complementary pathways to study size and number. These complementary techniques must be compatible with SP ICP-MS and offer non-destructive characterization capabilities to study particles before they are annihilated in the ICP. In a recent study,^19^ we have demonstrated the possibility of coupling a two-dimensional optical trap to SP ICP-MS. This allowed advanced characterizations of both inorganic particles and MPs. In that study, the two-dimensional trap using optofluidic force induction (OF2i) was used in a new mode, which enabled static trapping of particles in a weakly focused vortex laser beam. The interested reader will find further information on OF2i elsewhere.^21–23^ The combination of optical and fluidic forces enabled the trapping of up to 30–50 particles at size- and refractive index-dependent positions, and consequently, if the refractive index is known or determined, sizes can be calibrated using Mie theory. We could further show that appending OF2i with an SP Raman module enabled the identification of the polymer type at single MP resolution.^19^ The hyphenation of optical traps and mass spectrometric techniques has a high potential to advance MP analysis by combining their advantages and avoiding some of their limitations.

In this study, we advance from previously demonstrated dual couplings to the first functional realization of an online trimodal platform that unifies OF2i, SP Raman, and SP ICP-MS. The newly developed prototype enables optical trapping, molecular identification, and elemental analysis within a single analytical workflow. Using the OF2i static trapping mode, we employ an optical extraction mechanism to isolate suspended particles from complex matrices, thereby reducing the background and enabling species-specific analysis. Moving beyond model standards, we demonstrate how the developed platform addresses realistic analytical challenges, bypassing colloidal and dissolved interferences and characterizing industrial primary MP feedstock polymers within complex environmental matrices.

## Materials and Methods

### Consumables and Sample Preparation

PS standards (5 μm) were obtained from Sigma-Aldrich (St. Louis, MO, USA). PA-6 was provided by BASF SE (Dx50 = 7.5 μm). Na_2_CO_3_ (≥99.5%, p.a., ACS, anhydrous) was purchased from Carl Roth and used to create a 1 g/L dissolved C standard. For the first proof-of-concept, this standard was spiked with PS MPs. 100 nm Au nanospheres (100 nm) were bought from NanoComposix (San Diego, CA, USA) and a 10 ng/mL ionic Au standard was diluted from a commercial single element ICP-MS standard in pure water. Ultrapure water was obtained from a Merck Millipore (18.2 MΩ·cm, Merck Millipore, Bedford, USA) system and used for dilutions and washing steps if not indicated otherwise. For dilutions and storage, 50 mL polypropylene vials were used. The MP background from these vials was investigated by preparing and analyzing blanks via both OF2i and SP ICP-MS, and no MPs were found.

The preparation of the soil sample followed an extraction protocol developed by Pfohl et al.^24^ 2 g of autoclaved soil (Lufa 2.2, LUFA Speyer, Germany) were spiked with 75 mg of PA-6. To improve the dispersibility and deagglomeration of the microplastic and soil particles, one droplet of the surfactant Lutensol TO7 (BASF SE) was added. The recovery of MPs was approximately 70% and more details on the extraction method can be found in the respective protocol.^24^ A blank soil sample without a PA-6 spike was treated equally in parallel.

### Instrumentation

A Brave B-Curious OF2i instrument using a cylindrically shaped microfluidic flow channel (1.3 mm) was used and operated with a 532 nm CW DPSS laser with a max power of 2 W (Laser Quantum, GEM532). Beam alignment was performed using two mirrors and a 5× beam expander. Using a zero-order vortex half-wave plate (*q* = 1), an azimuthally polarized Laguerre–Gaussian laser mode with a topological charge of *m* = 2 was generated and focused within the flow cell. Light scattered by particles was magnified and recorded with an ultramicroscope setup. The Raman signal of single particles was resolved using a prism and recorded at a 90° angle using a CMOS camera. The frequency shift was calibrated into wavenumbers by using an internal calibration approach in which the Raman signals of water stemming from the aqueous media were used as anchor points. The spectra of detected MPs were compared to reference spectra of common polymers. While there was a high correlation between the Raman signal of MPs and reference spectra, some intensity differences were apparent for different bands. These differences were the result of the wavelength of the applied laser, which was significantly lower than the wavelength used in established Raman spectrometers and used for reference spectra recording.

An Agilent 7900 series (Agilent Technologies) ICP-MS instrument was used in SP mode using a dwell time of 0.1 ms and acquiring the ^12^C signal for 2 min intervals. H_2_ was used as reaction/collision gas at between 2 and 3 mL/min as discussed in a previous study.^18^ A total consumption nebulizer and spray chamber (CytoNeb, CytoSpray, Elemental Scientific, Omaha, US) were used to increase aerosol transport efficiency, which was determined to be between 60 and 80% by analyzing 100 nm and a 10 ng/g Au standard while maintaining a flow rate of 8 μL/min using a peristaltic pump. The same flow rate was used when coupling OF2i, SP Raman spectroscopy, and SP ICP-MS.

### Data Analysis

The OF2i instrument was operated with HANS software (Brave Analytics), and a live video feed was recorded at 200 fps. SP Raman data were recorded at 7.7 fps and analyzed using in-house developed MATLAB routines.

SP ICP-MS data was recorded with MassHunter (Agilent Technologies) and saved as .csv files. Subsequently, SPCal was used for further data processing.^25,26^ For the calculation of mass and size of a detected MP event, ^12^C signals were calibrated using the mean mass response of a previously analyzed 5 μm PS standard. An iterative thresholding algorithm was used, which removed SP signals based on a preliminary threshold to find the mean value of the background signal. This was repeated until no particle events were found anymore. At high mean values (>10 cts), Gaussian statistics (5σ) were used, and Poisson statistics (α = 10^–8^) were used otherwise. Found SP signals were accumulated above the mean signal value, and the resulting peak area data array was exported for subsequent graphical analysis via OriginPro (OriginLab 8.5).

## Results and Discussion

### Hyphenation of OF2i, SP Raman, and SP ICP-MS

In a previous proof-of-concept,^19^ we suggested a new hyphenated technique, which combined SP ICP-TOFMS with OF2i to trap and characterize particles optically before atomization in the ICP. We further demonstrated that OF2i can be coupled with an SP Raman module gaining chemical selectivity to identify MP types at single particle resolution. In this study, we advance on the previous set-ups and hyphenate all three techniques online. This combined certain advantages while eliminating some of the limitations. The resulting trimodal platform combining OF2i, SP Raman, and SP ICP-MS (see Figure 1) provided new analytical avenues to manipulate and characterize MPs in complex environments. Figure 1A shows a scheme of the optical trap, which uses a laser vortex beam to trap several particles according to their size and refractive index via OF2i. Harnessing both optical and fluidic forces in a 2D optical trap provides critical advantages when compared to other optical trapping systems. Compared to a Gaussian beam setup, several particles can be trapped statically in a size and refractive index-orientated order. Furthermore, the vortex beam presents a “donut” shape, which allows trapping of particles within the same size and refractive index range next to each other while minimizing particle–particle interactions within the trap.

**Figure 1:**
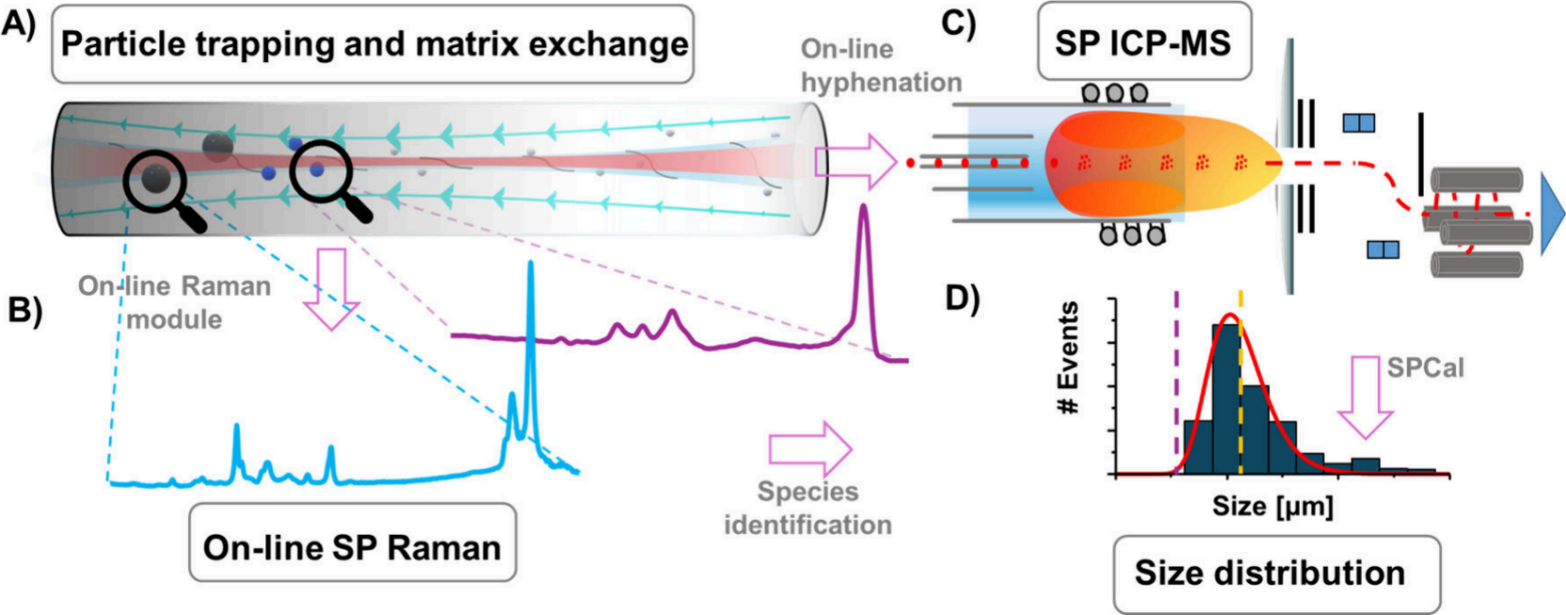
Trimodal instrumental set up. (A) OF2i using a 2D optical trap and a weakly focused vortex beam with angular momentum. (B) Inelastically scattered laser light of trapped particles was analyzed via Raman spectroscopy. (C) SP ICP-MS was coupled online after the trap and, following the release of particles, used to detect MPs via the ^12^C signal. (D) SP signals were processed with SPCal to estimate size distributions of detectable MPs.

Figure 1A and B show the SP Raman analysis of the statically trapped particles. The static trapping of particles allowed continuous recording of spectra for improved signal-to-noise ratios and lower detection limits. The Raman signal of single particles depended on size and refractive index. Therefore, the ability to detect small particles was influenced by both the polymer type as well as interferences from prominent Raman signals from the surrounding media (in this case aqueous media). For the MP species analyzed in this study, Raman-based size detection limits were estimated to be between 600 and 800 nm. However, it is worth noting that Raman spectroscopy considers only the inelastically scattered light fraction, which was significantly less intense than the elastically scattered fraction (Rayleigh scattering). When focusing on the latter, no species information could be obtained, but the position of substantially smaller particles could be analyzed as shown in Figure 2. Here, PS standard particles with sizes between 100 and 800 nm are shown and were size calibrated as demonstrated in a previous study.^19^ The top part demonstrates a scheme of the weakly focused vortex beam, and below is a photograph of the Rayleigh scattering of trapped particles. A round capillary was used and acted as a cylindrical lens shaping the scattered light of each particle to a line. The lateral position in the trap depended on the refractive index and the size of a particle. Therefore, for a known particulate polymer, sizes could be calibrated when considering optical forces (Mie theory) and drag forces.^19,21,23^

**Figure 2:**
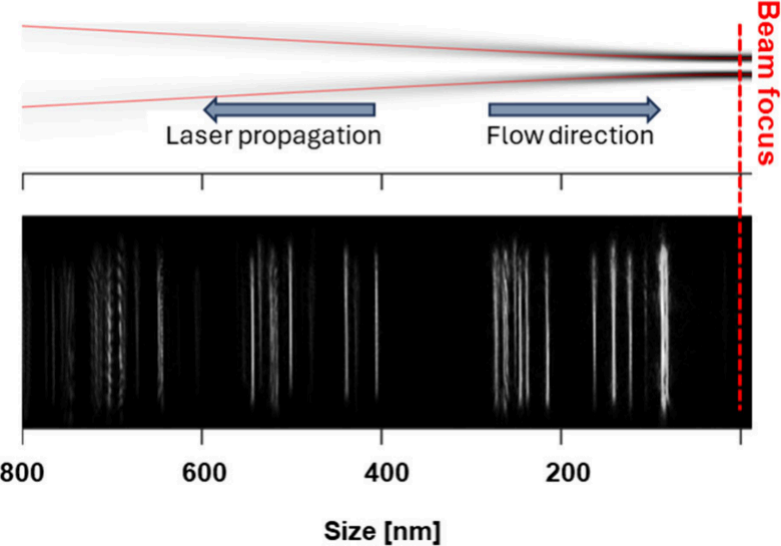
PS particles with sizes between 100 and 800 nm are statically trapped using OF2i. Shown is the Rayleigh scattering of particles and their lateral, size-dependent positioning within the optical trap.

Figure 1C illustrates ICP-MS instrumentation, which used a quadrupole mass analyzer. The hyphenation of OF2i-Raman with SP ICP-MS was achieved using a total consumption nebulizer and spray chamber setup, which provides high transport efficiencies at low sample flows. For this study, this was practical to maximize the number of detectable particles in SP ICP-MS when releasing and transferring particles from OF2i-Raman to SP ICP-MS instrumentation. The upper size detection limit was set by a decreasing transport efficiency of larger particles and was estimated to be around 10–15 μm, which was in line with a recent study by Fazzolari et al.^20^ As such, SP ICP-MS can only consider a fraction of MPs, and calibrated sizes refer only to “detectable” MPs while assuming spherical geometry. While the latter was accurate for PS MP standards, this cannot be assumed for secondary and aged MPs, and as such, size histograms for detectable particles are tentative. However, in this study, the focus was the development of an optical extraction mechanism to isolate particles, identify their polymer backbone, and exchange the matrix to improve detection limits in SP ICP-MS. Altogether, this enabled an online molecular and elemental characterization as shown in the following section.

### Optical Extraction of Microplastics

In SP ICP-MS, transient data comprise contributions from the dissolved fraction of an element, appearing as an elevated background, and from single particle events, appearing as short signal spikes with durations of typically 400–800 μs. The background shows a certain level of noise, which can be modeled with either Gaussian or Poisson statistics depending on the mean value. At increasing mean signal level, absolute noise levels increase as well, and the signal of small particles can be determined with less certainty. Especially for common elements, the dissolved background signal can be high and significantly deteriorate the detection limit. For MPs, interferences as well as high levels of dissolved C species complicate single event detection and decrease the analyzable size window significantly. This is a severe constraint for the analysis of small MPs in environmental matrices, which may contain a complex composition with various dissolved C species.

Here, we propose to use the optical trap to separate MPs from their initial matrix and to exchange the media with ultrapure water. This not only improved size detection limits in SP ICP-MS but also decreased the matrix burden and, consequently, matrix effects in the plasma. PS MPs (5 μm) were spiked into a solution containing 1 g/L of dissolved C (as Na_2_CO_3_). Subsequently, the sample was introduced into the optical trap, and PS particles were statically trapped as explained before (as shown for example in Figure 2). The maximum trapping capacity was around 30–50 particles, and when approaching this value, the carbonate matrix was exchanged with ultrapure water. SP ICP-MS was coupled online with the optical trap, and the transient SP data sets are shown in Figure 3A. Here, trace (a) shows the analysis of the high carbonate matrix, and some SP signals of a larger MP fraction can be seen. The lower size and mass detection limits were determined to be 3.2 μm and 18 pg carbon per particle during starting conditions, as shown in Figure 3B. During the extraction process (trace (b)), the carbonate matrix was replaced with ultrapure water reducing the background signal for C. During the replacement step of the matrix, particles were kept in the trap and no further MP events were detected in SP ICP-MS after an initial washing period. Trace (c) shows the dissolved background 40 min after initiating the matrix exchange process indicating a low and steady baseline, which resulted in a size and mass detection limit of 1.0 μm and 0.6 pg carbon per particle. The factors of improvement were 3.1 and 28 for size and mass limits during this proof-of-concept, respectively. Subsequently, extracted particles were released (trace (d)) for SP ICP-MS with enhanced detection limits. Previously trapped MPs were detected across approximately 2 min. It is noteworthy that the exchange process, transfer, and recovery of MPs via SP ICP-MS took several minutes, which was likely related to inefficient particle transfer between OF2i and SP ICP-MS, the requirement to run SP ICP-MS with low flows to maintain consistent transport efficiency, as well as several connections presenting dead volumes and sources of turbulence. In future studies, it may be possible to improve transfer time and reduce MP spread with dedicated microfluidic set-ups. This would translate into the possibility of cycling trapping and release experiments several times in short intervals to increase particle counting rates.

**Figure 3:**
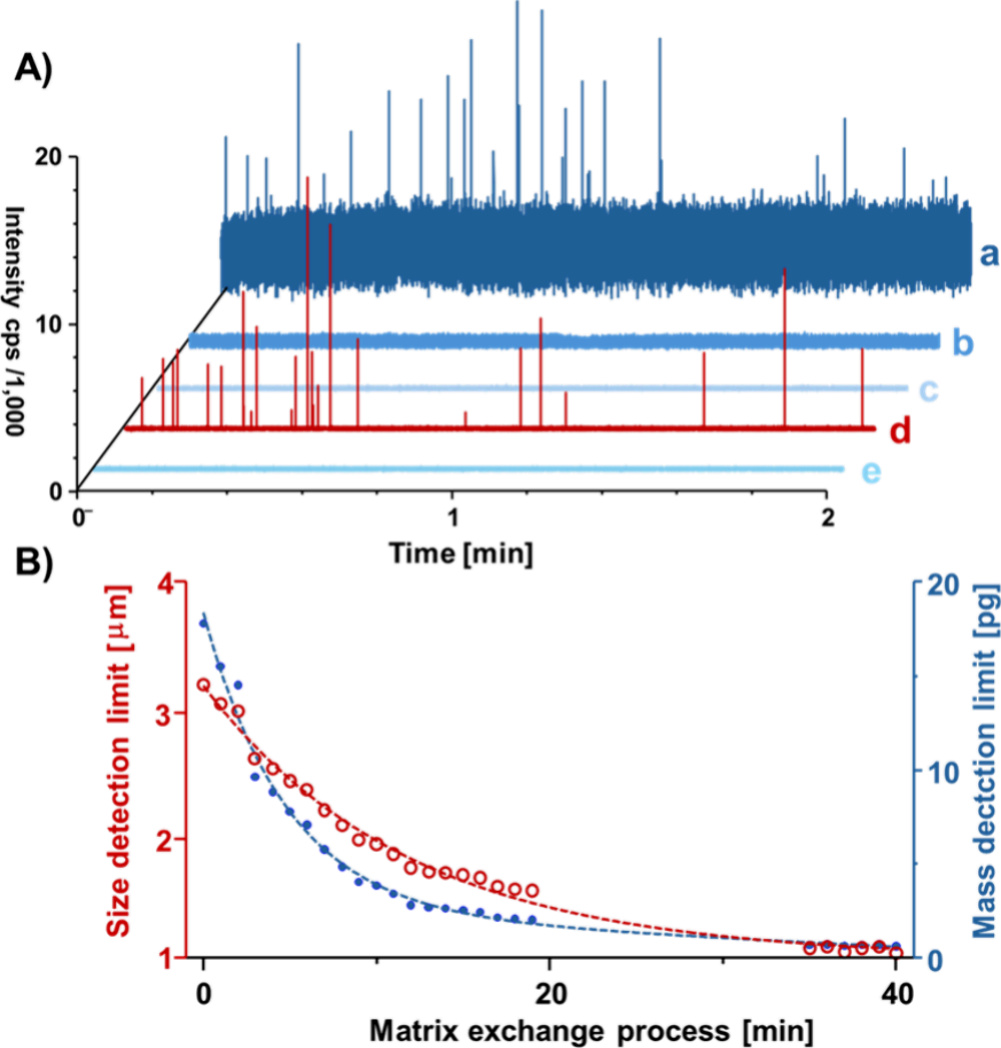
(A) Transient SP signals across different background levels. MPs were dispersed in a high carbonate matrix and trapped. Residual (nontrapped) particles can be seen with a high dissolved C background in trace (a). Following trapping, the carbonate matrix was replaced with ultrapure water (trace (b)) to reduce the dissolved C background and to improve mass and size detection limits. Trace (c) shows the background before releasing the trapped particles, which were detected in trace (d). Trace (e) shows the signal directly after the released particles were detected. (B) Size and mass detection limits for MPs across the matrix exchange process.

As OF2i was coupled not only with SP ICP-MS but also with a SP Raman module, it was further possible to access polymer information during trapping and extraction procedures. In environmental scenarios, the MP polymer is often not known, and the ability to identify it provides a critical advantage. On the one hand, knowledge of the MP type is important to understand environmental and/or health impacts. On the other hand, it is relevant to deduce the mass fraction and density, which are two parameters required to estimate particle mass and size in SP ICP-MS.

The optical extraction of a PS standard was repeated several times until at least 60 particles were counted in SP ICP-MS for estimating the size distribution of detectable particles. Figure 5A shows the resulting size histogram next to a representative single particle PS Raman spectrum. In the same diagram, a reference spectrum for PS (gray) is shown, demonstrating the capability to identify MPs without *a priori* knowledge.

### Analysis of Microplastics in Spiked and Extracted Soil

Following the first proof-of-concept with a PS standard, a soil sample spiked with irregular PA microplastics was analyzed for a second proof-of-concept simulating a more environmentally orientated scenario, in which MPs showed a larger polydispersity as well as a range of different shapes. During sample preparation, MPs were recovered using a resuspension and density separation protocol by Pfohl et al.^24^ This produced a suspension that also contained other colloidal matter and presented a scenario that is expected for most environmental samples, in which MPs represent only a small number-based fraction of the overall particulate matter. For these environmental scenarios, pinpointing and characterizing single MPs is challenging. Following dilution (1:10) of this suspension, PA-6 MPs were trapped optically, while other (smaller) colloidal matter was discarded. In this case, only PA-6 MPs were trapped, which facilitated further analysis via SP ICP-MS. In cases where other particulates are trapped next to MPs, OF2i-SP Raman characterization is still possible, but recovering the distinct particle species in SP ICP-MS is challenging. Figure 4A shows the Raman channel, in which the signal of four trapped PA-6 MPs can be seen. The Raman signals of water are located above 3000 cm^–1^, around 1640 cm^–1^, and below 250 cm^–1^, which was useful for internal calibrations of wavenumbers. The horizontal dimension represents the lateral position in the optical trap, and the intense scattering signal at the bottom was the Rayleigh scattering from MPs. The frequency shift caused by inelastic scattering of trapped PA-6 MPs can be seen along the vertical axis (e.g., yellow area). In the area (turquoise area in Figure 4A) behind the laser focus (red dashed line), no static trapping of MPs was possible, and the Raman signal was only caused by the aqueous media. This area was recorded to investigate the matrix composition during matrix exchange, and spectra recorded across three different times during the matrix exchange process are shown in Figure 4B. A signal at 950 cm^–1^ was caused by the matrix and was monitored as the indicator for when the matrix was exchanged, which was after approximately 10 min. After this time, the laser power was reduced to release the optically extracted particles from the trap. Figure 4C shows the calibrated Raman channel of one trapped particle, and the distinct Raman signal between 1100 and 1650 cm^–1^ was used to identify the MP polymer as PA-6. Following optical extraction, only PA-6 Raman signals were observed, and no other C particulates were retained. An unspiked soil was prepared accordingly as blank and no MPs were detected. Particles were subsequently recovered and analyzed by SP ICP-MS and the trapping, extraction, and release procedure explained for PS was repeated to estimate size distributions via SP ICP-MS as shown in Figure 5B. The mean size of detectable PA-6 MPs was 2.6 μm, and the size detection limit was determined to be 1.2 μm.

**Figure 4:**
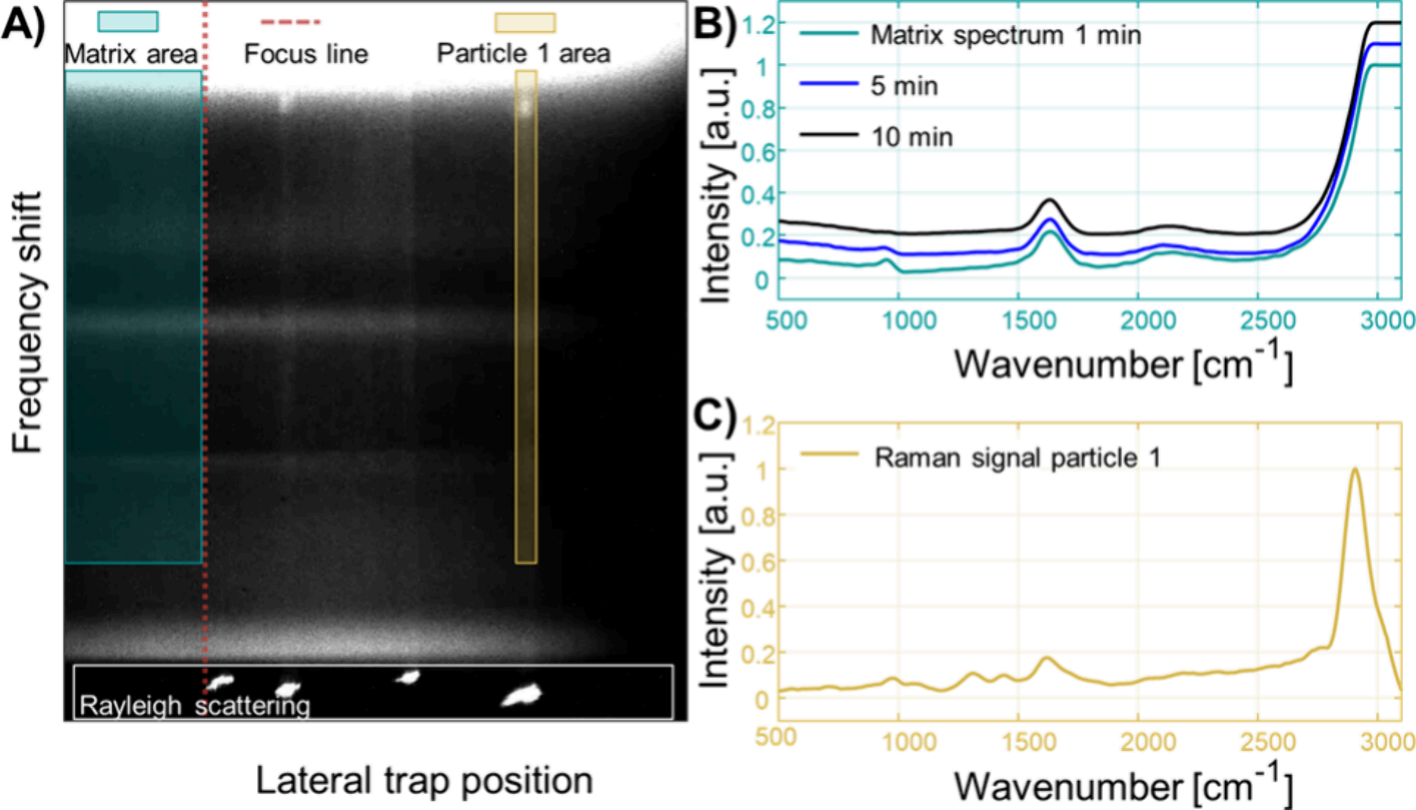
(A) Cutout of the Raman channel. The horizontal axis shows the lateral position of particles in the optical trap, and the four intense dots (bottom) correspond to the Rayleigh scattering of four trapped MPs. Along the vertical axis, the frequency shift due to inelastic scattering is shown, which was calibrated into a Raman spectrum. (B) Raman spectrum of the matrix sampled behind the focus line at three different time points after the matrix exchange process. The band at 950 cm^–1^ was caused by the matrix and was used as an indicator when the matrix exchange process was complete. The other bands corresponded to the Raman signal of water. (C) Raman spectrum of a single particle, which was corrected for the signal contribution of the water matrix. The three bands between 1100 and 1650 cm^–1^ are typical for PA-6 MPs.

**Figure 5:**
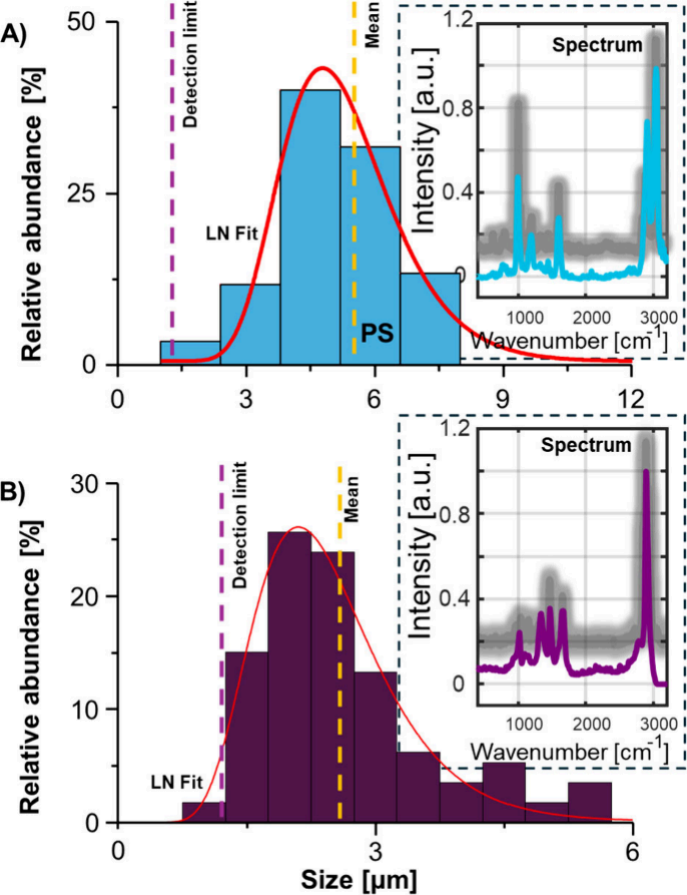
(A) The size distribution of detected PS MPs was estimated with SP ICP-MS. A selected Raman spectrum of a single PS MP is shown in the inset, and a PS reference spectrum is shown in gray. (B) The size distribution of extracted and detected PA-6 MPs was estimated with SP ICP-MS. A selected Raman spectrum of a single PA-6 MP is shown in the inset, and a PA-6 reference spectrum is shown in gray.

## Conclusion

The analysis of MPs challenges analytical techniques as they are required to address various chemical and physical facets, such as the chemical composition and sizes of particles in complex matrices. SP techniques are taking an important place to address the heterogeneity of particles and to describe both the internal and external mixing state of particles, while counting and characterizing MPs. However, current SP techniques alone provide only limited access to complex particulates, and new perspectives can be gained when combining different SP detection paradigms. In this study, a trimodal hyphenated SP platform consisting of OF2i, SP Raman, and SP ICP-MS has been developed for the first time to enable a multimodal analysis of individual MPs. A focus was directed to test the concept of optical extraction, which enabled the isolation, manipulation and characterization of MPs. This optical extraction mitigated matrix effects, enabled MP identification, and enhanced detection limits for MPs in SP ICP-MS as demonstrated in two proof-of-concepts.

While packages of particles can be trapped, released, and recovered by SP ICP-MS, it was not possible to trace the same particles between OF2i-Raman and SP ICP-MS. These limitations may complicate analyses when more than one MP polymer or other C particulates are present as their trapping order is not conserved when reaching the ICP. As such, it is not yet possible to gain both chemical and elemental information on the very same particles. However, using more efficient microfluidic transfer systems and for example an optical chromatography setup, which gradually reduces laser power to release single particles one-by-one, may render this possible in the future. Furthermore, Mie theory can be used in the future to provide complementary insights into size distributions, and OF2i may be used to count MPs and determine number concentrations. This provides opportunities not only for intrinsic validation of particle sizes but also to expand the analyzable size window and improve particle number calibrations.

Overall, this study moves beyond earlier dual-coupling feasibility tests to deliver the first operational online OF2i–SP Raman–SP ICP-MS platform. The system combines optical trapping, chemical characterization, and elemental analysis within a single workflow and provides opportunities for new extraction methods for particles. This made it possible to target relevant industrial microplastic particles in environmentally relevant matrices. However, it is worth noting that OF2i–SP Raman–SP ICP-MS is not limited to the analysis of MPs but may further be applied to the analysis of a range of inorganic (e.g., mineral) particles across the nano- and lower microscale, promising opportunities to gain new perspectives on relevant mineral-based particulates.
